# Bioactive Low Molecular Weight Keratin Hydrolysates for Improving Skin Wound Healing

**DOI:** 10.3390/polym14061125

**Published:** 2022-03-11

**Authors:** Laura Olariu, Brindusa Georgiana Dumitriu, Carmen Gaidau, Maria Stanca, Luiza Mariana Tanase, Manuela Diana Ene, Ioana-Rodica Stanculescu, Cristina Tablet

**Affiliations:** 1 SC Biotehnos SA, 3–5 Gorunului Street, 075100 Otopeni, Romania; lolariu@biotehnos.com (L.O.); dbrandusa@biotehnos.com (B.G.D.); luiza.craciun@biotehnos.com (L.M.T.); diana.ene@biotehnos.com (M.D.E.); 2 Academy of Romanian Scientists, 3 Ilfov Street, 030167 Bucharest, Romania; 3 Leather Research Department, National Institute for Textiles and Leather Division Leather and Footwear Research Institute (ICPI), 93 Ion Minulescu Street, 031215 Bucharest, Romania; maria.stanca@icpi.ro; 4 Horia Hulubei National Institute of Research and Development for Physics and Nuclear Engineering, 30 Reactorului Str., 077125 Magurele, Romania; istanculescu@nipne.ro; 5 Department of Physical Chemistry, University of Bucharest, 4–12 Regina Elisabeta Bd., 030018 Bucharest, Romania; cristinatablet@yahoo.com; 6 Faculty of Pharmacy, Titu Maiorescu University, 16 Gh. Sincai Bd., 040317 Bucharest, Romania

**Keywords:** keratin hydrolysate, bioactive keratin, skin homeostasis restoration, skin wound healing

## Abstract

Keratin biomaterials with high molecular weights were intensively investigated but few are marketed due to complex methods of extraction and preparation and limited understanding of their influence on cells behavior. In this context the aim of this research was to elucidate decisive molecular factors for skin homeostasis restoration induced by two low molecular weight keratin hydrolysates extracted and conditioned through a simple and green method. Two keratin hydrolysates with molecular weights of 3758 and 12,400 Da were physico-chemically characterized and their structure was assessed by circular dichroism (CD) and FTIR spectroscopy in view of bioactive potential identification. Other investigations were focused on several molecular factors: α1, α2 and β1 integrin mediated signals, cell cycle progression in pro-inflammatory conditions (TNFα/LPS stimulated keratinocytes and fibroblasts) and ICAM-1/VCAM-1 inhibition in human vascular endothelial cells. Flow cytometry techniques demonstrated a distinctive pattern of efficacy: keratin hydrolysates over-expressed α1 and α2 subunits, responsible for tight bounds between fibroblasts and collagen or laminin 1; both actives stimulated the epidermal turn-over and inhibited VCAM over-expression in pro-inflammatory conditions associated with bacterial infections. Our results offer mechanistic insights in wound healing signaling factors modulated by the two low molecular weight keratin hydrolysates which still preserve bioactive secondary structure.

## 1. Introduction

Keratin can be extracted from keratin-rich materials using chemical (reduction, oxidation, hydrolysis, sulphitolysis), physical (steam explosion, microwave irradiation) or biological methods [1]. The properties of keratin such as amino acid content, molecular weight, thermal behavior and bioactivity depend on the extraction and preparation methods [2,3]. Biomaterials prepared from keratin extracted from wool or human hair showed excellent properties regarding biocompatibility and cellular proliferation abilities. These properties make wool keratin an excellent material for tissue engineering and drug delivery systems [4]. In vitro studies have shown that keratin has the ability to scavenge free radicals, similar to that of vitamin C and allows replacing cosmetic preservatives [5].

A study on the anti-inflammatory ability of keratin extracted from human hair showed that it has a more pronounced anti-inflammatory effect on monocytic cell line, than collagen or hyaluronic acid. The study showed that primary macrophage cells are altered when they are exposed to an immobilized keratin biomaterial surface and that these changes appear to target an anti-inflammatory phenotype. This phenomenon appears to be a function of the lower molecular weight of keratin, even if it is recognized that the mechanism of anti-inflammatory process is not fully understood [6]. Tachibana et al. showed also that due to the amino acid sequence with more carboxylic terminal groups, keratin is more effective than collagen in binding osteoblast cells and inducing osteogenesis [7]. Biomaterials based on keratin contain regulatory molecules that make them capable of functioning as synthetic extracellular matrices (ECM) and promoting nerve tissue regeneration due to their capacity to create fibronectin-like cell binding that facilitates cell adhesion [8,9]. Gao et al. conducted in vivo and in vitro studies on the ability of keratin to promote the regeneration of peripheral nerves. The in vitro study showed that it has neuroinducible activity and Schwann cells grown in keratin medium have a number of changes in terms of migration capacity, morphology and proliferation activity, with stimulating effect on neuronal axons extension. In vivo experiments have shown that keratin accelerates the regeneration of the axon in the early stages and has a beneficial effect for subsequent functional recovery [10].

Human keratin hair hydrogel efficiency for treatment of thermal or chemical wounds was proved by faster closure and no increase in wound size in the first four days of healing [11]. In the field of skin regeneration, there are several mechanisms working together to restore dramatically affected molecular processes. For example, cellular function is regulated during dermal repair flow by critical interactions between receptors and extracellular matrix proteins. Also, secretory immune cells release cytokines and growth factors (IL-1, IL-4, IL-6, IL-13, TGF-β, TNF) interrelated with structural proteins synthesis [12]. This kind of interactions leads to regenerative processes’ activation, including angiogenesis and scar remodeling [13,14]. Integrins are heterodimeric proteins from plasmatic membrane, involved in cellular responses to growth factors, and direct regulation of transcriptional programs, signaling cascades and activation mechanisms [15]. Over-expression of specific integrins directs wound healing, fibrosis and scarring. They are transmembrane structures with α and β subunits assembled to form functional receptors: α3β1 (receptor for laminin 332), α6β5 (hemidesmosome component, receptor for laminin 332), α2β1 (receptor for collagen and laminin, having a pivotal role for wound healing in vivo), αvβ5 (receptor for vitronectin); α9β1 (receptor for tenascin C) [16,17]. A correlation was reported between the level of β1 integrins and proliferation in the interfollicular epidermis [18]. Integrins are also required for an essential step of skin regeneration, namely fibroblast infiltration into the wound clot. Normal fibroblasts and granulation tissue fibroblasts express many types of integrins: α1β1, α2β1, α3β1, α5β1, α11β1, ανβ1, ανβ3, and ανβ5, in order to bind collagens, fibronectins and other blood clot components. For example, fibroblasts interact with fibrillar collagens via α1β1, α2β1, and α11β1 integrins, regulating matrix metalloproteinase (MMP) expression and collagen fibrillogenesis [19]. Another event accompanying skin injuries is inflammation, including cytokines (IL6, IL8, TNFα) signals progression and cellular factors over-expression. Key elements for vascular endothelial inflammation are proteins from CAM family, especially ICAM (intercellular adhesion molecule) and VCAM (vascular cell adhesion molecule) [20]. They play a different role, namely: ICAM-1 specifically participates in trafficking of inflammatory cells, in leukocyte effectors’ functions, in adhesion of antigen-presenting cells to T lymphocytes, in microbial pathogenesis, and in signal transduction pathways through outside-in signaling events [21], and VCAM-1 that is one of the major regulators of leukocyte adhesion and transendothelial migration by interacting with α4β1 integration, which in turn activates intracellular signaling that allows transendothelial migration of leukocytes [22].

In our paper we hypothesized that the wool keratin, an easily available byproduct, can be tuned by chemical-enzymatic hydrolyses in different keratin biomolecules so as to stimulate the main skin cell biochemical mechanisms for wound healing. As in many reported research studies, the human hair keratin was extracted and solubilized through complex reduction or oxidizing methods and prepared in hydrogel forms mainly with high molecular weights [23], the present research proposes alkaline-enzymatic hydrolysates with lower molecular weights, prepared by complete wool solubilization [24] and with preserved bioactive structured molecules.

It is recognized that few commercial products based on keratin reached the market of wound healing biomaterials as compared to other materials due to the complex methods of preparation and the insufficient understanding of cellular interaction with keratin [25,26]. In this regard the present research proposes keratin hydrolysates prepared by a green, reproducible and easy method of solubilization [24] and brings evidences in several molecular factors, decisive for restoring the skin homeostasis as new potential wound dressing active component.

If different keratin dressings with high molecular weights were recognized as activators of keratinocytes wound healing process [27], the present research brings mechanistic insights from damaged skin cells restitution processes induced by two low molecular bioactive keratin hydrolysates with still structured peptides.

Thus, the stimulation effect on β1 glycoprotein expression in pro-inflammatory conditions and α1 and α2 subunits generation in the basal state of fibroblasts were proved by low molecular keratins. The other anti-inflammatory factors involved in wound healing process, like significant down regulation of nonspecific stimulation—TNFα + PMA, and the ICAM expression were also influenced.

According to our knowledge no similar study was performed regarding the influence of low molecular wool keratin hydrolysates on skin cells homeostasis factors.

## 2. Materials and Methods

### 2.1. Materials

Raw wool was purchased from a local sheep farmer (Lumina, Constanta, Romania). Chemical reagents of analytical grade like sodium hydroxide (98%), ammonia (25%), sodium carbonate (99.7%), formic acid (85%) and sulfuric acid (92%) were purchased from Chimopar Trading SRL (Bucharest, Romania). Borron SE (ethoxylated alkyl derivatives with 65% concentration) was supplied by SC Triderma SRL (Bucharest, Romania). Esperase^®^ 8.0 L, a serine endo-peptidase from *Bacillus lentus* with activity of 8 KNPU-E/g, working at elevated temperature and pH = 8–12.5, was purchased from Novozymes (Atasehir, Turkey). Valkerase^®^, a keratinase, serine protease from *B. licheniformis,* with activity of 80,549 U/g at pH = 5.5 and 55 °C, was supplied by BioResource International (Durham, NC, USA).

### 2.2. Keratin Hydrolysates Preparation

The alkaline hydrolysate was prepared by a method previously described [24]. Briefly, to obtain the alkaline hydrolysate, wool was pretreated by washing and degreasing using 4% *w*/*w* NH_4_OH, 0.6% *w*/*w* Borron SE and 1% *w*/*w* Na_2_CO_3_ for 2 h at 40 °C, rinsed to neutral pH and minced with a bench grinder machine (La Minerva, Minerva Omega, Bologna, Italy). After this treatment, wool was mixed with 2.5% *w*/*v* sodium hydroxide solution in a stainless-steel vessel equipped with a mechanical stirrer and automatic temperature control (SC Caloris SA, Bucharest, Romania) at 80 °C, for 4 h. The enzymatic keratin hydrolysates were obtained from alkaline hydrolysate by enzymatic hydrolysis using 1% *w*/*w* concentration of two enzymes: Esperase or Valkerase under optimum activity conditions for 4 h. The enzymatic keratin hydrolysates were conditioned at pH = 7 with sulfuric acid. The enzymatic keratin hydrolysates were labeled Ker 1 and Ker 2. Keratin hydrolysates were centrifuged for 15 min at 6000 rpm (Eppendorf 5804, Wien, Austria) and lyophilized by freeze-drying of keratin dispersions in a DELTA 2-24 LSC Freeze-dryer, laboratory scale (Osterode am Harz, Germany).

### 2.3. Keratin Hydrolysates Characterisation

Physical-chemical characteristics were evaluated according to the standard in force or in house methods for: dry substance (SR EN ISO 4684:2006), ash (SR EN ISO 4047:2002), total nitrogen and protein content (SR EN ISO 5397:1996), aminic nitrogen (ICPI method), cysteine (SR 13208:1994), cystine sulphur (SR13208:1994) content, and pH (STAS 8619/3:1990). The results were expressed as the average of triplicate determinations with standard deviations. The molecular weights were determined by gel permeation chromatography (GPC) using an Agilent Technologies instrument (1260 model) (Agilent Technologies, Santa Clara, CA, USA) equipped with PL aqua gel-OH MIXED-H column (7.5 × 300 mm × 8 µm) and multi-detection unit. Optimum working conditions for GPC were flow rate of mobile phase containing 1 mL min^−1^, injection volume of the sample 100 µL, and temperature of 35 °C for the detectors and column. Calculations of the Mw and number average molecular weight (Mn) were performed with the Agilent GPC/SECS software (Version 1.1, Agilent Technologies, Santa Clara, CA, USA). The free amino acid analysis was performed using a GC-MS (Thermo Scientific TRACE1310/TSQ 8000Evo, Waltham, MA, USA). Samples were derivatized with acetonitrile and BSTFA at 105 °C. The GC separation was achieved using a capillary column TG-5SILMS (5% diphenyl/95% dimethyl polysiloxane), 30 m × 0.25 mm × 0.25 µm. The operating conditions for GC were an initial temperature of 100 °C raised to 170 °C then to 190 °C (3 °C/min) and the final temperature was 280 °C. The total run time was 40 min. The temperature for the transfer line was set at 280 °C and the ionization source at 230 °C. Injection volume was 0.5 µL. The MS detector was operated in continuous scan mode with the *m*/*z* interval ranging from 40 to 600 amu. The data were analyzed using the Chromeleon v.7.2.7 software. For quantification of amino acids, a calibration curve was used.

Circular dichroism spectra were measured with a Jasco J-815 spectropolarimeter (Cremella, Italy) in 190–250 nm range, at room temperature in a 0.05 cm optical path quartz cuvette. The experimental parameters were set as follows: 1 nm bandwidth, 1 nm data pitch, 4 s response, 50 nm/min scanning speed, 3 accumulations. The sample solutions were prepared in a pH 7.4-buffer phosphate by diluting the stock solutions to 30 μM for Ker 1 and 121 μM for Ker 2. The normalized root mean square error deviations of deconvolutions were 0.02158 for Ker 1 and 0.02603 for Ker 2.

FTIR spectra were acquired in the 4000–400 cm^−1^ spectral range with a Bruker Vertex 70 instrument (Bruker, Ettlingen, Germany) with the following working parameters: 4 cm^−1^ resolution, 0.1 cm^−1^ wavenumber accuracy, 0.1% T photometric accuracy and 64 scans. A KBr beam splitter and a RTDLaTGS (Room Temperature Deuterated Lanthanum α Alanine doped TriGlycine Sulphate) detector were used. The measurements were performed by the technique of pastillation in KBr of spectroscopic purity (Merck reagent) at a KBr: sample weight ratio of 300:1 toward a 300 mg KBr reference disc. The discs were prepared by pressing at 10 t/cm^2^, under vacuum, the fine powder obtained after grinding for 5 min in an agate mortar. Spectra were processed with the OPUS software (Bruker, Ettlingen, Germany) using atmospheric compensation, vector normalization, baseline correction with straight lines and one iteration additional concave rubber band correction and automatic peak peaking. The deconvolution parameters were settled in the spectral range of 1230–1330 cm^−1^ using Gaussian functions generated by ORIGIN 9 software. FTIR deconvolution data residual RMS error was less than 0.001.

The morphology of lyophilized enzymatic keratin hydrolysates was analyzed by scanning electron microscopy with a FEI Quanta 200 Scanning Electron Microscope (FEI, Eindhoven, The Netherlands) with a gaseous secondary electron GSED detector at an accelerating voltage of 12.5–20 kV.

### 2.4. Biocompatibility Tests

To establish the dose effect relation of lyophilized keratins, as well as the cytocompatibility conditions, standardized cell lines specific to the dermo-epidermal layer and vascular endothelium were used as follows: Human dermal Fibroblast (normal cell line HS27—ATCC^®^ CRL-1634™) cultivated in DMEM (Dulbecco’s Modified Eagle’s Medium /Nutrient Mixture F-12 Ham, Sigma-Aldrich (Saint Louis, MO, USA) supplemented with 10% fetal bovine serum (Sigma-Aldrich) and 1% Antibiotic Antimycotic Solution (100×) (Sigma Aldrich); Normal Human Keratinocytes (immortalized cell line HaCaT purchased from ThermoFisher Scientific) cultured in Dulbecco’s Modified Eagle’s Medium (DMEM) (ATCC^®^), with high content of glucose, supplemented with 10% fetal bovine serum and 1% Antibiotic Antimycotic Solution (100×) purchased from Sigma Aldrich; Human Umbilical Vein Endothelial Cells (HUVEC—CRL-1730™) cultured in RPMI-1640 media, supplemented with 10% fetal bovine serum, 1% L-glutamine, and 1% Antibiotic Antimycotic Solution (100×) purchased from Sigma-Aldrich. All cell lines were cultured or incubated at 37 °C with 5% CO_2_.

Determination of the cytotoxic profile was performed correlating cell viability monitored by intracellular esterase activity (labeling with tetrazolium salt MTS using the CellTiter 96^®^AQueous One Solution Cell Proliferation Assay (Promega)) with release of lactate dehydrogenase in culture medium due to affected cell membrane permeability (LDH test using the CytoTox 96^®^ Non-Radioactive Cytotoxicity Assay Kit (Promega)). The cells were allowed to adhere for 24 h (7000 cells/well) and treated for 48 h with the test substances. The staining was done according to the protocol for the specific reagent kit (MTS/LDH). All concentrations of active principle were tested in triplicate and the data were acquired using an ELISA TriStar 941 multimode plate reader from Berthold Technologies (Bad Wildbad, Germany).

For the specific, wound-healing activity investigation, several experimental series were processed, in the following cycle of cultivation: 24 h for adhesion + 48 h for physiological development and incubation with tested compounds. Some cells were grown under normal conditions, other cells were activated overnight either with 15 ng/mL TNFα and 0.1 μM PMA (phorbol myristate acetate), to mimic acute inflammation, associated with pro-oxidative conditions, or with 1 μM suspension of LPS (bacteria lipopolysaccharide) to mimic bacterial infection.

Cell cycle sequential analysis was performed by flow cytometry according to Cycle Test Plus DNA Reagent kit (BD Pharmingen) protocol, which involves dissolving the cell membrane lipids with a nonionic detergent, removing the cytoskeleton and nuclear proteins with trypsin, enzymatic digestion of cellular RNA and stabilization of nuclear chromatin with spermine. Propidium iodide is stoichiometrically bound to purified DNA and the complex formed is analyzed by flow cytometry [28].

The integrin detection technique by flow cytometry involves the use of monoclonal antibodies for α and β chains from BD Pharmingen (CD49a, fluorescent labeled for PE—corresponding to α2 integrin; CD49b, fluorescent labeled for FITC, corresponding to α1 integrin; and CD 29 fluorescent labeled for APC, corresponding to integrin β1). Results were compared with the specific isotype control (PE Mouse IgG1, k Isotype control, APC Mouse IgG1, k Isotype control, FITC Mouse IgG1, k Isotype control). Analysis of the vascular anti-inflammatory effect was performed by simultaneous fluorescent labeling with the appropriate antibodies for ICAM-1 and VCAM-1, respectively. The cell suspension is labeled with APC Mouse Anti-Human CD54 for ICAM-1 (intracellular-adhesion molecules) and with PE Mouse Anti-Human CD106 for highlighting VCAM-1 (vascular-cell-adhesion molecules). Flow cytometry results concerning membrane molecules stained with fluorescent antibodies were compared with the specific isotype controls (PE Mouse IgG1, k Isotype control, APC Mouse IgG1, k Isotype control, FITC Mouse IgG1, k Isotype control).

**Figure 1:**
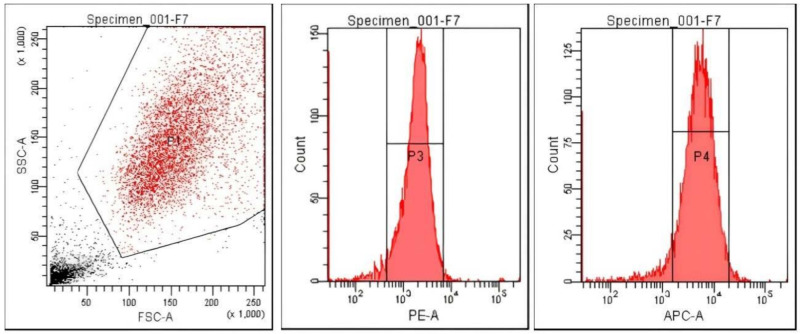
Dot-plot of cellular population and histograms corresponding to ICAM-1 (APC-A) and VCAM-1 (PE-A) signals.

Acquisition and analysis were done with FACS CANTO II (Becton-Dickinson) and FCS Express software—DNA cell cycle module and DIVA 6.1 software. Figure 1 presents an example of flow-cytometry diagram concerning fluorescent antibody staining of membrane proteins.

### 2.5. Statistical Analysis

The statistical processing was done using analysis of variance (ANOVA) (95% significant level) on each pair of interest and differences at *p* < 0.05 were considered statistically significant.

## 3. Results

### 3.1. Keratin Hydrolysates Characteristics

The main physical-chemical characteristics of the two keratin hydrolysates in powder form are presented in Table 1. It can be seen that Ker 1 has lower molecular weight in correlation with higher aminic nitrogen concentration and lower cysteine and cystinic sulphur due to the higher breaking degree of keratin molecules. The polydispersity and free aminoacids concentrations are very similar. Molecular weights of 10,000–30,000 Da are known to be considered low molecular weights for keratin extracts [29] and according to our knowledge, the behaviour of keratin extracts under these values related to their structure and properties in interaction with different cell lines was not reported.

**Figure 2:**
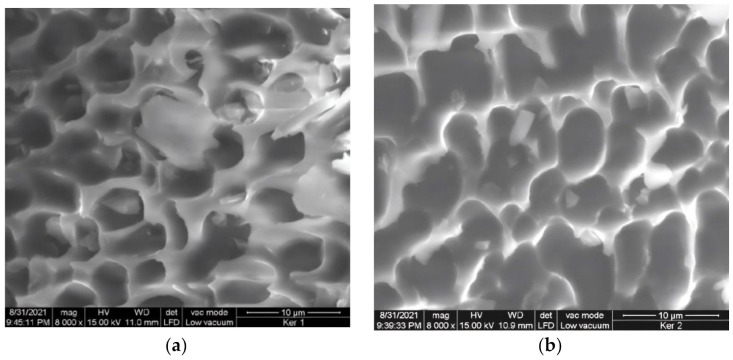
SEM images of lyophilized keratins: (**a**) Ker 1 and (**b**) Ker 2.

The morphology of lyophilized keratins is presented in Figure 2 and showed that the powders have a porous, interconnected structure.

### 3.2. Circular Dichroism Spectroscopy Analyses (CD)

**Figure 3:**
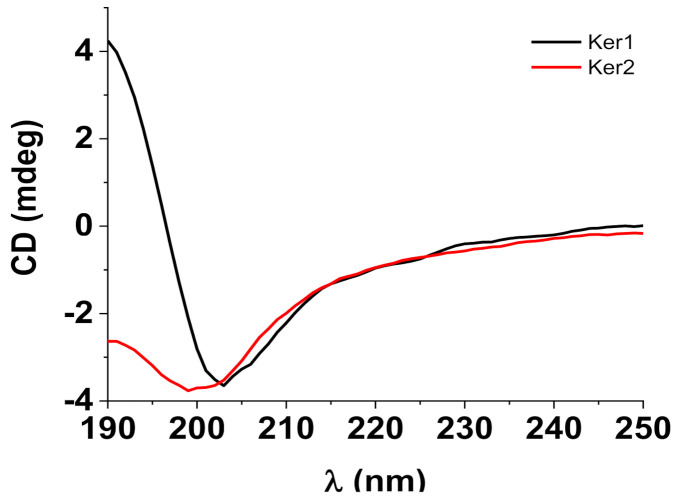
Circular dichroism spectra of lyophilized keratins.

Circular dichroism (CD) is widely used to gain information about a protein’s secondary structure. Thus, the presence of the α-helix in the protein structure is reflected in the CD spectra by the existence of three bands: a negative band at 208 nm and a positive one at ~195 nm due to π-π* transitions and a 220 nm negative band due to n-π* transitions. A quick look at the spectra from Figure 3 suggests low α-helix content for both samples. For a quantitative analysis of the secondary structure, we have used the BeStSel webserver [30,31]. The analysis results are displayed in Table 2.

### 3.3. Fourier Transform Infrared Spectroscopy (FTIR) Analysis

**Figure 4:**
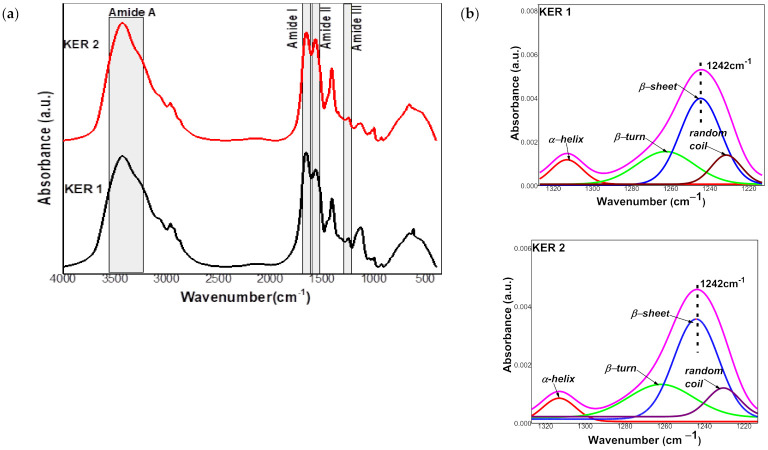
(**a**) FTIR spectra of Ker1 and Ker2; (**b**) Amide III band deconvolution of Ker1 and Ker 2.

Due to the variation of the protein content, molecular weight and conformation of Ker 1 and Ker 2, important changes can be observed in the FTIR spectra highlighted in the Figure 4a, regarding the main protein bands, Amide A, Amide I, Amide II and Amide III, their shape and intensity [32].

The Amide I band is due to the stretching vibrations of the C=O and C–N groups in the peptide bonds and is correlated with the backbone conformation. The Amide II band is associated with N–H plane deformation vibrations and C–N and C–C tensile vibrations and is conformationally sensitive. The Amide I/Amide II increased ratio of Ker 1 is correlated with an increase in total ordered structures content as seen in Table 3 [33,34]. Ker 1 has an intense band between 1105 and 1140 cm^−1^ which indicates the presence of oxidized cysteine and Bunte salt [32]. The Amide A band is correlated with N–H stretching vibrations and is an indicator of the hydrogen bonds formed between polypeptide chains leading to twisted sheet structures. The shift of the Amide A band of Ker2 to a higher wavenumber indicates a weaker hydrogen bond network in correlation with lower molecular weight (Table 1). Data from the literature show that the deconvolution of the Amide III band (Figure 4b) allows the reliable quantification of the secondary structures using the characteristic peaks of α-helix (1330–1295 cm^−1^), β-sheet (1250–1220 cm^−1^), β-turn (1295–1270 cm^−1^) and random coil (1270–1250 cm^−1^) [35,36]. Thus, the secondary structure of the Ker 1 additive consists of 49.8% ordered structures, while the Ker 2 additive has a slightly lower proportion of ordered structures, respectively 46.2% (Table 3).

### 3.4. Cytotoxicity Determination

**Figure 5:**
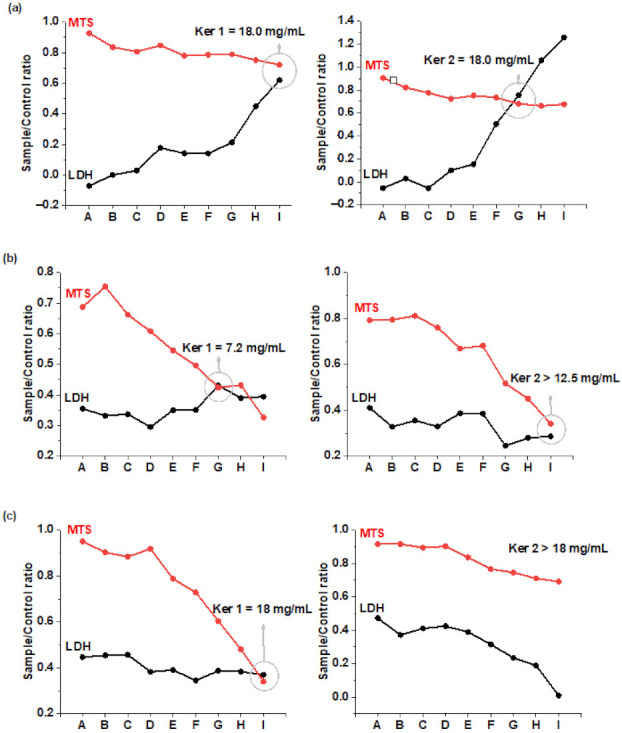
Cytotoxic profile of Ker 1 and Ker 2 on: (**a**) dermal fibroblasts (HS27); (**b**) keratinocytes (HaCaT); (**c**) endothelial cells (HUVEC). The sample concentrations were: A: 0.6 mg/mL, B: 3.0 mg/mL, C: 6.0 mg/mL, D: 9.0 mg/mL, E: 10.2 mg/mL, F: 12.0 mg/mL, G: 13.2 mg/mL, H: 15.0 mg/mL, I: 18.0 mg/mL.

The effect of keratin hydrolysates on three cell lines viability (MTS test) and their cytotoxicity (LDH test), respectively, was plotted as the ratio of test sample concentrations to the corresponding control and is presented in Figure 5.

In Figure 5 can be seen that the intersection point between the curves of the two analyzed parameters (LDH and MTS), represents the dose of the signal for the amount of lactate dehydrogenase released and exceeds the signal for the amount of MTS transformed into formazan, being equivalent to the dose at which cell viability is significantly affected. In Table 4 the maximum doses allowed for Ker 1 and Ker 2 are presented for different cell lines. It can be seen that Ker 2 has higher maximum doses for two cell lines as compared to Ker 1 which can be attributed to lower molecular weight with more available carboxylic groups [7].

### 3.5. Specific Activity of Lyophilized Keratins on Epidermal, Dermal and Vascular Endothelial Cells

**Figure 6:**
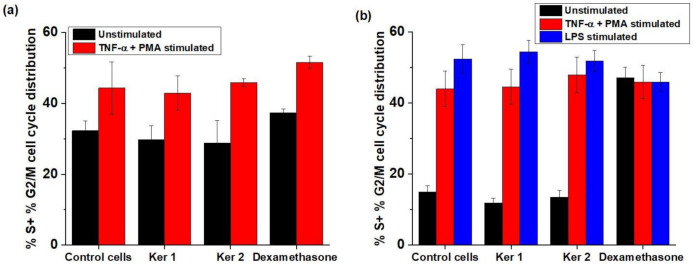
Mitotic phases’ evolution modulated by the lyophilized keratins: (**a**) HS27, (**b**) HaCaT (differences between cells treated with Ker1, Ker2 and positive control were done using ANOVA test—95% significant level on each pair of interest and differences at *p* < 0.05 were considered statistically significant).

In the healing of skin wounds the combination of the two mechanisms concurred: regeneration (affected cells are “de novo” replaced) and repair (the scar tissue reconstruction). The process by which fibroblasts and keratinocytes migrate to the site of the wound is replicated by mitosis to form a thin film of cells interconnected by adhesion molecules, growth factors and structural proteins, playing a key role in proper healing, as we previously described. This study’s investigations focused on the analysis of several molecular factors with a decisive role in restoring skin homeostasis: the dermo-epidermic proliferative status on HS27 and HaCaT cells, α1, α2 and β1 integrin mediated signals, and ICAM-1/VCAM-1 inhibition in human vascular endothelial cells (HUVEC). In order to simulate the inflammation associated with skin lesions, the unstimulated cells were compared with the pro-inflammatory state induced by TNFα + PMA or LPS stimulation, respectively. The positive control was considered dexamethasone, a potent anti-inflammatory drug. Results regarding the proliferative status of keratinocytes and fibroblasts, expressed as cell cycle progression are presented in Table 5 and Table 6 and Figure 6.

The fibroblasts’ mitotic phases’ evolution has no significant change after keratin hydrolysates’ treatment, but Ker 1 and Ker 2 increased the keratinocytes’ percent of nuclei in S-phase (DNA replication), Ker 2 being more active than Ker 1.

**Figure 7:**
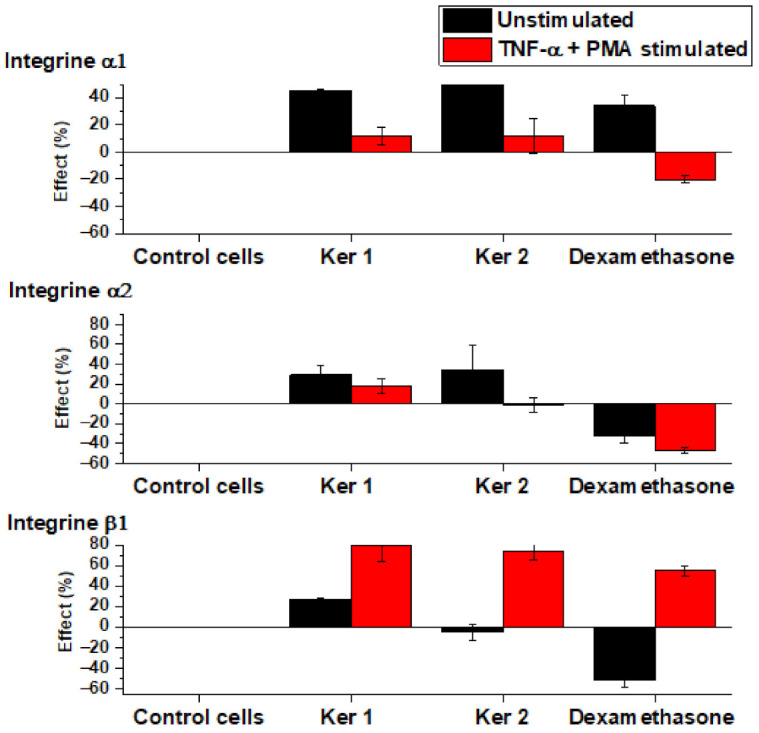
Percentage variation of integrins in fibroblasts compared to control cells for integrins α1, α2 and β1 over-expression modulated by Ker 1 and Ker 2.

Cell adhesion, essential for tissue repair process and mediated by specific interactions of integrins with extracellular matrix, was investigated through membrane over-expression of α1, α2 and β1 integrins in fibroblasts. Flow cytometry data presented in Table 7 and Figure 7 revealed a significant contribution of both types of keratins on stimulation of β1 glycoprotein expression in pro-inflammatory conditions, and higher presence of α1 and α2 subunits in the basal state of fibroblasts induced by Ker1 and Ker2. The influence of low molecular keratin on cell communication and function was revealed for hair keratin gel in the first stage of wound healing process when stronger regulatory ability was highlighted [29]. The same study stated the relation between keratin molecular weight and wound healing properties is still unknown.

Cell adhesion, essential for tissue repair process and mediated by specific interactions of integrins with extracellular matrix, was investigated through membrane over-expression of α1, α2 and β1 integrins in fibroblasts. Flow cytometry data presented in Table 7 and Figure 7 revealed a significant contribution of both types of keratins on stimulation of β1 glycoprotein expression in pro-inflammatory conditions, and higher presence of α1 and α2 subunits in the basal state of fibroblasts induced by Ker 1 and Ker 2.

**Figure 8:**
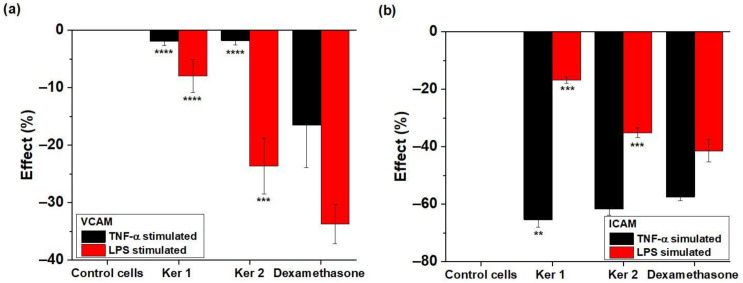
Percentage variation of (**a**) VCAM and (**b**) ICAM expression modulated by Ker1 and Ker2 compared to the cell control. ** *p* < 0.01, *** *p* < 0.001, **** *p* < 0.0001.

Inflammation associated with skin wounds reflected the expressions of CAM-family proteins at vascular endothelial cells’ level (VCAM and ICAM), with role in monocytes’ adhesion to endothelium. The experimental results obtained on pro-inflammatory conditions simulating acute systemic disregulation (TNFα + PMA) and bacteria aggression (LPS) are presented in the Table 8 and Figure 8. Data reflects the vascular endothelium membrane expression of ICAM-1 and VCAM-1 in fixed cells stained with fluorescent specific antibodies and acquired by flow–cytometry.

The bacterial attack at the endothelial level (stimulation with LPS) is counteracted by the Ker1 and Ker2 which significantly reduce the expression of ICAM (particularity for small blood vessels), as well as VCAM (specific to large blood vessel). Ker 2 showed increased efficiency in ICAM and VCAM expression reduction for LPS stimulation. In the case of nonspecific stimulation—TNFα + PMA, the ICAM expression is significantly down-regulated compared to the positive control (dexamethasone) for both keratins.

## 4. Discussion

Keratin extracted from sheep wool or human hair showed a high potential for wound healing biomaterials design [37,38,39], but the methods for keratin solubilization are very sophisticated and the mechanisms of cellular response are not completely understood [26]. The present research proposes the keratin hydrolysates with low molecular weights as wound healing additive, prepared by complete solubilization of sheep wool and by enzymatic refinery of molecular weight with still preserved secondary structure and showing bioactivity in integrin signaling, cell cycle modulation and vascular-cell-adhesion molecules inhibition.

The morphology of lyophilized keratin powders shows porous and interconnected architecture that represents a premise for cell proliferation, adhesiveness favoring the cell migration and wound healing [40]. The porosity of biomaterials is considered a critical property for cell migration due to the potential of facilitating oxygen, nutrition and metabolism exchange [41].

Circular dichroism investigations demonstrated that the solutions of keratin hydrolysates Ker 1 and Ker 2, with low molecular weights, still preserve the secondary structures of native keratin which represent the premise for their biocompatibility and cell biostimulation of wound healing mechanisms [42].

The high content in cysteine which generates very stable cystine bridges make keratin one of the most stable proteins with low solubility, which is difficult to extract. As compared to the reported methods for keratin hydrolysate extraction known as harsh methods that lead to the loss of natural function of protein, the present research showed that native structure is still preserved with effect on keratin immunogenicity which is much reduced as compared to collagen [43]. Recently reported research showed that the enzymatic route of wool keratin preparation is a new approach for obtaining high molecular keratin, with self-assembling properties for tissue engineering, but with disordered molecular structures [40], as compared to the present research which approaches methods based on chemical-enzymatic hydrolyses allowing to develop still organized structures with bioactivity in skin cell regeneration.

Ker 2 in solution has a larger percent of β-sheet organized molecules than Ker 1, meanwhile Ker 2 shows to have a parallel β-sheet fraction of 7.4%, probably due to the self-assembly ability of proteins with more ionic side groups and lower molecular weight [2]. The higher ratio of β sheet/β turn of 2.964 for Ker 2 as compared to Ker 1 with 1.785, showed improved biocompatibility [44], in correlation with higher cytotoxicity concentration for 2 cell lines (Table 3), more efficiency in keratinocytes increase and higher activity in ICAM and VCAM reduction in LPS stimulation test.

Ker 1 with a superior molecular weight and higher concentration of α-helix secondary structure is more active than Ker 2 on the binding site of integrins, both in normal and pro-inflammatory conditions, restoring the dermal strength through fibroblasts—collagen/laminin/fibronectin interactions.

FTIR spectra showed the shift to higher wavenumber of the Amide A band of Ker 2 indicating a weaker network of hydrogen bonds due to the presence of more side functional groups and lower molecular weight. Amide III deconvolution of keratin powders has revealed higher proportion of α-helix, 11% and 9.2% respectively, as compared to keratin solutions with 5.8% and 4.7% respectively, calculated from circular dichroism analyses which suggest a potentially slightly higher biocompatibility [45] in powder form. The results are similar with reported results for human hair keratin hydrolysate, with 12.18% α-helix attributed to the extraction conditions which favoured the conversion of native α-helix into β-sheet structures [46].

The behaviour of keratin hydrolysates in solution showed a tendency of developing more β-sheet and β-turn organized structures (41.9% and 48.1%, respectively) as compared to powder forms (38.8% and 37%, respectively). The β sheet/β turn ratio values for keratin powders has very similar values of 0.434 for Ker 1 and 0.464 for Ker 2, respectively. Similar values were reported in the case of wool keratin extracted by oxidative methods and with molecular weight of 13–31 kDa [47]. It can be concluded that keratin hydrolysates in solution showed a higher ability to organize and to generate β sheet and β turn secondary structures with a higher ability for lowest molecular weight Ker 2, as compared to powder form for which, α-helix ratio is higher for Ker 1, with higher molecular weight.

The biocompatibility studies performed through correlative methods for metabolic activity and cellular toxicity revealed a very good skin tolerance for higher doses of keratin preparations, expressed at all significant cellular exponents for wound healing: standardized cell lines HaCaT—human immortalized keratinocytes, HS27—human normal dermal fibroblasts and HUVEC—primary vascular endothelium from human umbilical vein.

Our studies also demonstrated a homeostasis restoring role of hydrolyzed keratin forms on skin inflammatory disruptions, injury associated. Ker 1 and Ker 2 presented a differentiated action at epidermal, dermal and vascular level, regulating significant molecular targets of the healing process. Inflammation was induced by external stimuli such as molecules from bacterial infection—lipopolysaccharide (LPS), originating from outer membrane of Gram-negative bacteria but also by molecules produced in several signaling cascades [48]. We chose tumor necrosis factor alpha (TNFα), a pro-inflammatory cytokine that triggers the expression of inflammatory molecules, including cell adhesion molecules—intercellular cell adhesion molecule-1 (ICAM-1) and vascular cell adhesion molecule-1 (VCAM-1) [22]. Compared with unstimulated series, the in vitro experimentally-induced pro-inflammatory signals were effective in key factors modulation and appropriate for the investigation of compounds effects in the wound healing context. Integrin-mediated adhesion is a bidirectional event in order to modulate the extracellular matrix structure and composition as well as to decide cell fate during wound healing through complex pathways: proliferation, differentiation, migration [19]. One of the most important integrins is β1 integrin (fibronectin receptor), which mediates the cellular response such as adhesion, migration, extracellular matrix assembly and signal transduction [49]. The presented screening showed a marked influence of keratin hydrolysates, Ker1 being stronger than Ker2, on β1 integrin, especially in pro-inflammatory conditions. Results gain thus relevance for the rapid relief of skin injuries after keratin treatment. In addition, Ker1 and Ker2 have a strong affinity for α1 and α2 subunits in the basal state of fibroblasts, preserving the essential cell—collagen interactions. The endothelium plays an important role in inflammation by regulating vascular permeability for macromolecules and leukocytes, vascular tone and hemostasis, and by producing and binding inflammatory mediators [50]. Skin injury activates adhesion molecules VCAM-1 and ICAM-1 in endothelial cells, and triggers the subsequent release of chemokines and inflammatory cell infiltration. Ker1 and Ker2 are involved in this factor’s membrane expression, after their up-regulation during endothelial activation. We have to mention the complex but unspecific action on both ICAM-1 and VCAM-1 when the inflammatory phenotype was induced by bacterial LPS and the selective effect on ICAM-1 when the systemic cytokines signals (TNF α) are involved.

Further research will be carried out for new formulations based on Ker1 or Ker2 and in vivo tests of their efficiency in wound healing.

## 5. Conclusions

The complex screening focused on essential mechanisms in delayed and difficult wound healing has demonstrated the involvement of two keratin hydrolysates with low molecular weights in skin regeneration processes.

The main conclusions of the study are that the lowest molecular weight keratin hydrolysate has a better cytotoxicity profile in dermo-epidermal cells and an anti-inflammatory effect on endothelial cells stimulated with LPS concerning the monocytes—endothelium adhesion molecules inhibition.

The study highlights correlations between the structural profile and the molecular weight of the two types of keratins, Ker1 and Ker2, and the modulation of certain signaling pathways in activating the post-traumatic recovery of the dermo-epidermal tissue. Thus, Ker1 (with superior molecular weight and higher concentration of α-helix secondary structure) is more active than Ker2 on the binding site of integrins, both in normal and pro-inflammatory conditions, restoring the dermal strength through fibroblasts—collagen/laminin/fibronectin interactions. In turn, Ker 2, better than Ker 1, accelerates the epidermal turnover, improving reepithelization probably due to the more available surface contact and biocompatibility induced by the higher ratio of β sheet/β turn structure. The slight impact of keratins on the proliferative status of fibroblasts has to be mentioned, which brings another therapeutical advantage, preventing tissue fibrosis and imperfect scarring. The obtained results contribute to the knowledge regarding mechanistic insight in wound repair, directing future medical applications based on low molecular hydrolyzed keratin preparations.

## Figures and Tables

**Table 1:** Physical-chemical characteristics of lyophilized keratin hydrolysates.

Characterization	Samples	
	Ker 1	Ker 2
Dry substance, %	96.91 ± 0.35	94.95 ± 0.32
Total ash, %	15.07 ± 0.24	14.26 ± 0.20
Total nitrogen, %	12.38 ± 0.34	11.68 ± 0.30
Protein substance, %	75.04 ± 0.34	70.78 ± 0.32
pH, pH units	7.27 ± 0.10	6.80 ± 0.10
Aminic nitrogen, %	0.89 ± 0.05	0.92 ± 0.05
Cysteine, %	9.03 ± 0.03	7.88 ± 0.03
Cystinic sulphur,%	2.41 ± 0.03	2.10 ± 0.03
Mw, Da	12,400	3758
Polydispersity index	1	1.05
Free amino acids,%	4.9	4.3

**Table 2:** Secondary structure percent composition obtained with BeStSel webserver for keratin samples.

Sample	α-Helix	ß Sheets		Turn	Others
		Antiparallel	Parallel		
		%			
Ker 1	5.8	27.5	0.0	15.4	51.3
Ker 2	4.7	25.5	7.4	15.2	47.2

**Table 3:** Secondary structure percent composition obtained from deconvolution of Amide III band of keratin powders.

Sample	α-Helix	ß Sheets	ß Turn	Random Coil
		%		
Ker 1	11.0	12.3	26.5	50.2
Ker 2	9.2	11.2	25.8	53.8

**Table 4:** The maximum dose allowed for Ker 1 and Ker 2 depending on the cell line tested.

No. crt	Sample	Cell Lines, mg mL^−1^		
		HS27	HaCaT	HUVEC
1	Ker 1	18	7.5	18
2	Ker 2	13.2	>12	>18

**Table 5:** HS27 proliferative status expressed as cell cycle progression in normal and pro-inflammatory conditions.

Tested Material	Unstimulated, %			TNF-α +PMA Stimulated,%		
	G0/G1	S	G2/M	G0/G1	S	G2/M
Control cells	69.22 ± 4.8	2.57 ± 0.2	29.69 ± 3.03	55.64 ± 7.4	18.99 ± 2.1	25.34 ± 5.3
Ker 1 (0.5 mg/mL)	70.20 ± 3.9 ***	2.10 ± 0.1 ***	27.70 ± 4.01	57.13 ± 4.8 ***	15.38 ± 3.7 ***	27.48 ± 1.1 **
Ker 2 (0.5 mg/mL)	70.55 ± 5.3 ***	2.60 ± 0.9 ***	26.18 ± 7.3 **	54.09 ± 1.1 ***	18.87 ± 1.4 **	27.03 ± 2.6 **
Dexamethasone	63.37 ± 1.5	5.90 ± 1.0	30.71 ± 2.5	48.40 ± 1.7	27.30 ± 1.3	24.27 ± 0.3

** *p* < 0.01, *** *p* < 0.001, using Repeated Measures ANOVA, Dunnett’s Multiple Comparison Test.

**Table 6:** HaCaT proliferative status expressed as cell cycle progression in normal and pro-inflammatory conditions.

Tested Material	Unstimulated, %		
	G0/G1	S	G2/M
Control cells	49.03 ± 2.3	4.86 ± 0.12	10.12 ± 1.01
Ker 1 (0.5 mg/mL)	48.14 ± 3.1	2.05 ± 0.6 ****	9.81 ± 0.8 ****
Ker 2 (0.5 mg/mL)	46.49 ± 1.6	3.42 ± 0.3 ****	10.09 ± 0.4 ****
Dexamethasone	52.93 ± 4.2	31.7 ± 0.4	15.37 ± 0.9
	**TNFα +PMA stimulated,%**		
	G0/G1	S	G2/M
Control cells	56 ± 0.92	32.59 ± 1.05	11.42 ± 0.12
Ker 1 (0.5 mg/mL)	55.4 ± 1.1	33.99 ± 0.9 ****	10.60 ± 0.6 **
Ker 2 (0.5 mg/mL)	52.05 ± 1.3	34.86 ± 1.9 **	13.09 ± 0.9
Dexamethasone	54.05 ± 1.6	30.99 ± 1.2	14.95 ± 0.7
	**LPS stimulated, %**		
	G0/G1	S	G2/M
Control cells	47.59 ± 4.7	39.56 ± 2.09	12.85 ± 1.6
Ker 1 (0.5 mg/mL)	45.53 ± 5.03 ***	43.02 ± 3.21 ****	11.44 ± 0.9
Ker 2 (0.5 mg/mL)	48.14 ± 3.21	41.90 ± 1.23 ****	9.92 ± 1.06
Dexamethasone	54.08 ± 4.78	34.47 ± 2.21	11.46 ± 0.75

** *p* < 0.01, *** *p* < 0.001, **** *p* < 0.0001, using Repeated Measures ANOVA, Dunnett’s Multiple Comparison Test.

**Table 7:** Membrane-specific fluorescence quantified through flow-cytometry showing integrins α1, α2 and β1expressed by fibroblast cells (HS27).

Tested Material	Integrin α1 (FITC-Mean-Relative Fluorescence Units)		Integrin α2 (PE-Mean-Relative Fluorescence Units)		Integrin β1 (APC-Mean-Relative Fluorescence Units)	
	Unstimulated	TNF α + PMA	Unstimulated	TNF α + PMA	Unstimulated	TNF α + PMA
Control cells	6611.0 ± 70.8	11,227.3 ± 716.2	2723.7 ± 215.5	12,599.3 ± 890.6	4831.7 ± 677.3	5427.3 ± 377.9
Ker 1 (0.5 mg/mL)	9601.0 ± 112.5 **	12553.0 ± 273.4 ***	3507.7 ± 152.3 ****	14,892.0 ± 1265.1 ****	6162.0 ± 923.4 ****	9677.7 ± 309.0 **
Ker 2 (0.5 mg/mL)	10,271.7 ± 1054.6 **	12,498.3 ± 647.7	3645.3 ± 398.6 ***	12,375.3 ± 1213.5	4543.7 ± 292.2 ***	9439.0 ± 204.2 **
Dexamethasone	8872.3 ± 413.7	8951.0 ± 875.0	1843.0 ± 46.9	6715.0 ± 829.1	2361.0 ± 560.8	8400.7 ± 571.2

** *p* < 0.01, *** *p* < 0.001, **** *p* < 0.0001, using Repeated Measures ANOVA, Dunnett’s Multiple Comparison Test.

**Table 8:** Adhesion molecules’ modulation by keratin hydrolysates in activated vascular endothelial cells (HUVEC).

Tested Material	VCAM (PE-A Mean-Relative Fluorescence Units)		ICAM (APC-A Mean-Relative Fluorescence Units)	
	LPS Stimulation	(TNF α+ PMA) Stimulation	LPS Stimulation	(TNFα+ PMA) Stimulation
Control cells	7428 ± 180.0	3516 ± 194.5	18,249 ± 507.7	13367 ± 751.1
Ker 1 (0.5 mg/mL)	6834 ± 88.5 ***	3448 ± 323 ****	15,168 ± 1768.7 ***	4612 ± 189.6 **
Ker 2 (0.5 mg/mL)	5677 ± 456.2 ***	3454 ± 327.14 ****	11,833 ± 130.9 ***	5122 ± 544.4
Dexamethasone	4921 ± 220.5	2912 ± 107.17	10,670 ± 533.5	5684 ± 330.5

** *p* < 0.01, *** *p* < 0.001, **** *p* < 0.0001, using Repeated Measures ANOVA, Dunnett’s Multiple Comparison Test.

## Data Availability

Data are contained within the article.
